# Modulation of serotonin signaling by the putative oxaloacetate decarboxylase FAHD-1 in *Caenorhabditis elegans*

**DOI:** 10.1371/journal.pone.0220434

**Published:** 2019-08-14

**Authors:** Giorgia Baraldo, Solmaz Etemad, Alexander K. H. Weiss, Pidder Jansen-Dürr, Hildegard I. D. Mack

**Affiliations:** 1 Department of Molecular and Cell Biology, Research Institute for Biomedical Ageing Research, University of Innsbruck, Innsbruck, Austria; 2 Center for Molecular Biosciences Innsbruck (CMBI), University of Innsbruck, Innsbruck, Austria; 3 Department of Biochemistry and Genetics of Aging, Research Institute for Biomedical Aging Research, University of Innsbruck, Innsbruck, Austria; Tokat Gaziosmanpasa University, TURKEY

## Abstract

Human fumarylacetoacetate hydrolase (FAH) domain containing protein 1 (FAHD1) is a mitochondrial oxalocatate decarboxylase, the first of its kind identified in eukaryotes. The physiological role of FAHD1 in other eukaryotes is still poorly understood. In *C*. *elegans* loss of the FAHD1 ortholog FAHD-1 was reported to impair mitochondrial function, locomotion and egg-laying behavior, yet the underlying mechanisms remained unclear. Using tissue-specific rescue of *fahd-1(-)* worms, we find that these phenotypic abnormalities are at least in part due to *fahd-1*’s function in neurons. Moreover, we show that egg-laying defects in *fahd-1(-)* worms can be fully rescued by external dopamine administration and that depletion of *fahd-1* expression induces expression of several enzymes involved in serotonin biosynthesis. Together, our results support a role for *fahd-1* in modulating serotonin levels and suggest this protein as a novel link between metabolism and neurotransmitter signaling in the nervous system. Finally, we propose a model to explain how a metabolic defect could ultimately lead to marked changes in neuronal signaling.

## Introduction

Human fumarylacetoacetate hydrolase domain containing protein 1 (FAHD1) is a FAH superfamily member which was recently identified in eukaryotes [1]. This protein family is commonly present in prokaryotes, where its members catalyze a broad variety of biochemical reactions [1, 2]. While several prokaryotic FAH proteins and the eponymous mammalian enzyme FAH are well-characterized, the enzymatic activity and physiological function of additional eukaryotic family members is still largely unclear. Human FAH-domain containing protein 1 (FAHD1) has been reported to be a mitochondrial protein that exhibits oxaloacetate decarboxylase activity *in vitro*, raising the interesting possibility that human cells possess a previously unrecognized way of reducing flux through the TCA cycle [3]. In particular, under physiological conditions, the TCA-cycle is supplied with carbon through the citrate synthase-catalyzed condensation of oxaloacetate and acetyl-CoA [4]. However, if FAHD1 is not expressed, metabolic flux through the TCA cycle may be altered, as in the liver and kidney of *Fahd1* knockout mice, oxaloacetate levels are significantly increased relative to wild type mice [3]. In a model derived from these findings, FAHD1 acts to fine tune TCA cycle flux in response to changes in metabolic activity [5].

In the nematode *Caenorhabditis elegans*, loss of *fahd-1* causes striking phenotypic alterations. Reduced oxygen consumption and decreased membrane potential in *fahd-1* deficient worms relative to wildtype [6] indicate that FAHD-1 is required for proper mitochondrial metabolism and may fulfill a role similar to that proposed for human FAHD1 [3]. Moreover, *fahd-1* deficient worms exhibit impaired locomotion and egg-laying behavior, suggesting that *fahd-1* modulates animal physiology at least in part through its activity in neurons and/or muscles. The mechanism controlling egg-laying in *C*. *elegans* have been dissected genetically and includes, among other regulators, the neurotransmitters serotonin and dopamine, which stimulate and repress, respectively, the release of eggs [7, 8]. Other processes regulated by serotonin and dopamine in *C*. *elegans* include backward and forward locomotion and male mating [9, 10]. Finally, studies on worms carrying an inactivation in the gene *tph-1*, which encodes a tryptophan hydroxylase involved in serotonin biosynthesis, indicated that serotonin signaling and fat metabolism are interconnected as they both respond to a common upstream signal [11].

Here, we sought to elucidate the molecular basis for the phenotypic defects in locomotion and egg-laying observed in *fahd-1(-)* worms [6]. We find that *fahd-1* modulates these behaviors at least in part through its function in neurons and provide evidence for *fahd-1* ensuring proper signaling through serotonin by increasing the expression of serotonin-biosynthetic enzymes. Given the high homology between nematode and mammalian FAHD-enzymes, it seems possible that human FAHD1 also regulates serotonin-dependent neurotransmission.

## Material and methods

### *C*. *elegans* strains and culture

Strains used in this study are listed in Supplementary S9 Table. The *fahd-1(tm5005)* allele, which comprises a large deletion and thus, is predicted to be a *null* allele (therefore referred to as *fahd-1(-)* hereafter), was obtained from the *National Bioresource Project for the experimental animal “Nematode C*. *elegans”* of Japan and outcrossed six times to our lab’s N2 wildtype strain to generate strain HMT059, which represents the *fahd-1(-)* condition in all experiments. *fahd-1*(-) genotype was verified by PCR-based genotyping as described (6). Worms were cultured following standard protocols [12] on NGM agar plates seeded with *E*. *coli* OP50 at 20 °C and synchronized for experiments by timed egg-laying for two hours unless stated otherwise.

### Generation of *fahd-1* rescue strains

Full body and tissue specific rescue strains were generated by microinjecting plasmids (20 ng/μl) containing the *fahd-1* genomic sequence fused to either the *fahd-1* promoter (defined as 2,020 bp immediately upstream of the ATG) or the *rab-3* promoter into the distal gonads of adult *fahd-1(-)* worms. p*myo-2*::*gfp* (50 ng/μl; plasmid pL4040, Addgene #1621, from A. Fire’s lab) served as a coinjection marker. The p*fahd-1*::*fahd-1* transgenic line was described previously [6]. The p*rab-3 fahd-1* plasmid was generated as follows: the *fahd-1* genomic sequence (corresponding to ZK688.3.1, including all exons and introns) was amplified from the p*fahd-1*::*fahd-1* plasmid and placed downstream of the *rab-3* promoter in plasmid pGH8—pRAB-3::mCherry::unc-54utr (Addgene #19359, from E. Jorgensen’s lab) instead of the mCherry sequence, using the restriction enyzmes XbaI and Eco52I. Following isolation of transgenic lines based on inheritance of the coinjection marker, *fahd-1* re-expression was confirmed by genotyping and Western Blot.

### Protein extraction and Western Blot analysis of FAHD-1 expression

For protein extraction, approximately 5,000 nematodes were washed off five freshly starved 6 cm plates with M9 buffer into a 1.5 ml Eppendorf tube and washed three additional times with 1 ml M9 buffer to remove excess *E*. *coli* OP50. Following the last wash, as much buffer as possible was removed and the remaining worm pellet was snap-frozen in liquid nitrogen. Subsequently, the pellet was placed on ice, re-suspended in 150 μl lysis buffer [6] and subjected to sonification (Sonifier 250 (Branson), 10 pulses, 1 sec each, stage 4). Protein concentration was determined by Bradford assay. 30 μg of total protein were separated on a 12% SDS gel and transferred to a PVDF membrane. After blocking in 5% milk solution in TBST for at least 1 hour, membranes were incubated with 2 μg/ml polyclonal rabbit anti-FAHD-1 antibody (produced by BioGenes GmbH, Berlin, Germany, using full-length His-tagged ceFAHD-1 protein produced in chick cells for immunization; purified in our lab from serum via NHS–column) or a monoclonal mouse antibody against the loading control β-actin (JLA20 Calbiochem CP01, 1:10,000, Calbiochem, La Jolla, USA) in 5% milk solution. Secondary antibodies and incubation conditions were polyclonal swine anti-rabbit HRP-conjugate (1:2,500, #P0399 Dako, Glostrup, Denmark) and polyclonal rabbit anti-mouse HRP-conjugate (1:10,000, #P0447, Dako). Signals were detected using enhanced chemiluminescence (Millipore, Billerica, USA) on X-ray films.

### Locomotion assay

Locomotion rate was quantitated by counting body bends as described in [6], following the protocol outlined in [13]. Synchronized day one adult worms were placed on individual non-seeded NGM agar plates and allowed to roam freely for one minute. The number of body bends performed during this time was recorded for at least 45 nematodes per strain and biological replicate.

### Egg-laying assay

Synchronized day one adult worms were placed into 50 μl M9 buffer in individual wells of a 96 well plate and inspected for egg-laying every hour over a period of four hours. At each time point, the total number of eggs laid by each worm up to this time point was counted for at least 45 worms per strain and biological replicate.

### Neurotransmitter exposure assay

Serotonin hydrochloride (Sigma, Vienna, Austria), fluoxetine (Sigma, Vienna, Austria), levamisole (Sigma, Vienna, Austria), and dopamine hydrochloride (Sigma, Vienna, Austria) were dissolved in M9 buffer at 5mM and 10mM, and 15mM, respectively. Day one adult worms synchronized by bleaching were placed into individual wells of a 96 well plate containing 50 μl of the appropriate serotonin or dopamine solution. The number of eggs laid by each worm was counted after two hours of incubation for at least 50 worms per strain, condition and biological replicate.

#### RNA isolation

Total RNA was isolated from approx. 1,200 day one adult worms synchronized by bleaching, with TRI Reagent (Sigma) and further cleaned up using RNeasy MiniElute Cleanup Kit (#74204, Qiagen, Hilden, Germany). RNA yield and quality was determined with a Nanodrop 2000 instrument (Thermo Scientific, Delaware, USA).

#### Primers for qPCR and Genotyping

Primers were designed using the *Primer-BLAST* program freely available at the NCBI webpage and synthesized by Eurofins Genomics (Ebersberg, Germany). Primer sequences are listed in S7 Table.

### Statistical analysis

Statistical analysis was performed using GraphPad Prism 5 software. All experiments were conducted in at least three biological replicates. Depending on the experiment, statistical significance was determined using Student´s t-test or ANOVA with Bonferroni post-tests to account for multiple comparisons.

## Results

### Full body re-expression of *fahd-1* restores locomotion in *fahd-1(-)* worms

**Fig 1:**
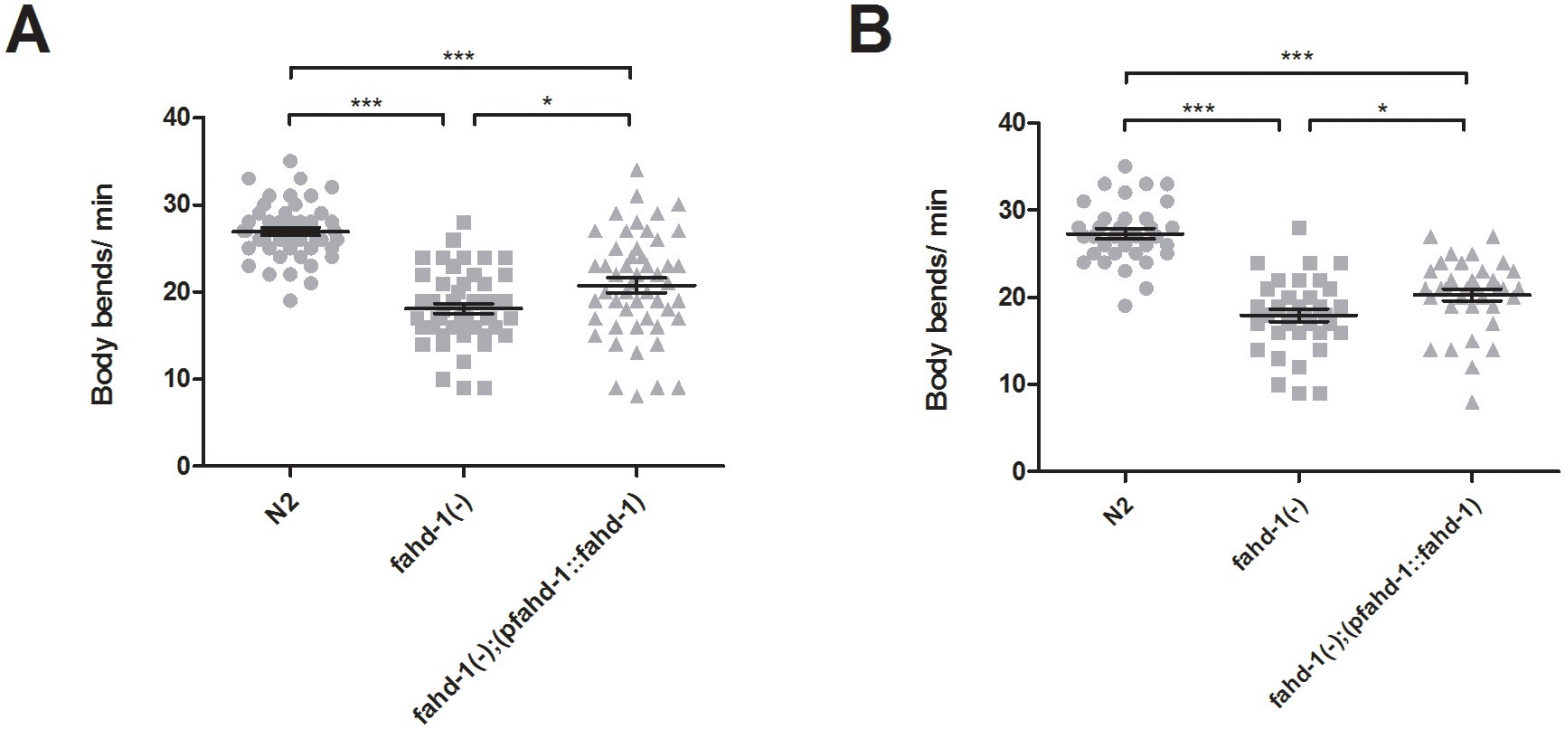
*Fahd-1* acts to control locomotion of *C*. *elegans*. *fahd-1* was re-expressed in the *fahd-1(-)* mutant background under its native promoter in the entire body and the mean number of body bends per minute across at least 35 day one adult worms per strain was determined. Panels A and B represent two independent transgenic lines. Error bars indicate standard errors. Statistical significance was determined by one-way ANOVA with Bonferroni post-tests. * and *** indicate p<0.05 and p<0.001, respectively, ns indicates no statistical significance. Data shown is combined from at least two independent experiments. See S1 Table for complete statistical analysis.

To begin to gain further insights into *fahd-1*’s physiological role, we re-introduced a full-length *fahd-1* wildtype gene under the control of the *fahd-1* native promotor back into *fahd-1(-)* worms and examined its effect on prominent *fahd-1(-)* phenotypes. The *fahd-1* native promoter drives *fahd-1* expression in a broad variety of tissues [6], and our p*fahd-1*::*fahd-1* transgene rescued, albeit only partially, the locomotion deficit of *fahd-1(-)* worms in three independent lines (Fig 1A and 1B; and data not shown). Interestingly, when the p*fahd-1*::*fahd-1* transgene was expressed in an *fahd-1(+)* background, locomotion was reduced to a level similar to that observed in *fahd-1*(-) worms (S1 Fig). Western Blot analysis further revealed an increased *fahd-1* level in the whole-body rescue strain compared to wildtype worms (S2 Fig). Together, these findings are consistent with the notion that *fahd-1* is required to ensure normal *C*. *elegans* locomotion, but exerts an inhibitory function on movement when overexpressed.

### Egg-laying in *fahd-1*(-) cannot be stimulated by exogenous serotonin

**Fig 2:**
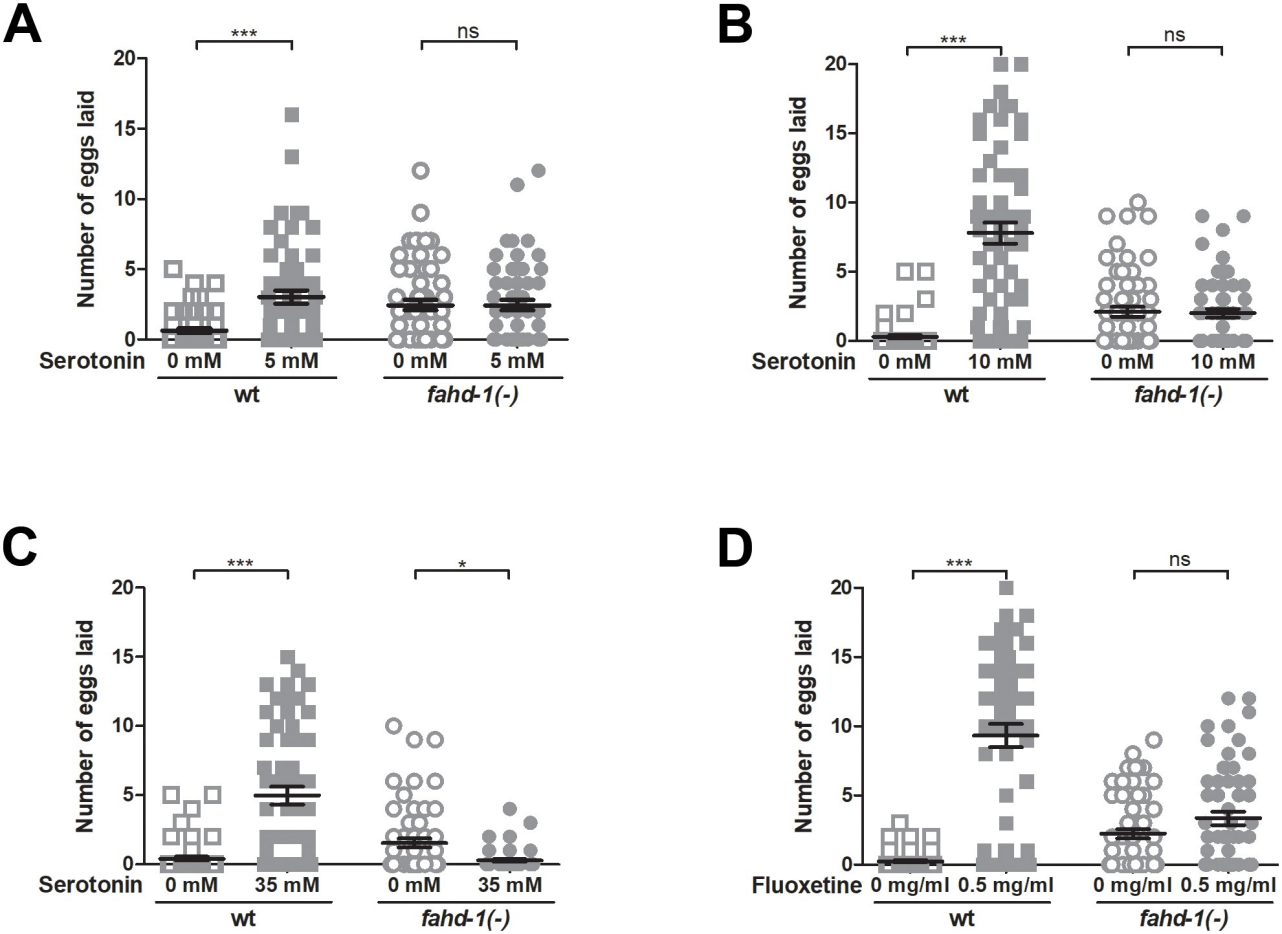
Effects of serotonin and the serotonin reuptake channel inhibitor fluoxetine on egg-laying behavior of *fahd-1*(-) worms. Wild type and *fahd-1(-)* worms were incubated in [A] 5 mM serotonin, [B] 10 mM serotonin, [C] 35 mM serotonin, or [D] 0.5 mg/ml fluoxetine, and the mean number of eggs laid after two hours was determined across at least 55 day one adult worms per strain. Error bars indicate standard errors. Statistical significance was determined by two-way ANOVA with Bonferroni post-tests. *** indicates p<0.001, * indicates p<0.05, ns indicates no statistical significance. Data shown is combined from 3–5 independent experiments. See S2 Table for complete statistical analysis.

Having established that *fahd-1* controls locomotion, and knowing from previous studies that egg-laying in *fahd-1*(-) is decreased [6], we asked whether these functions of *fahd-1* involved modulation of signaling through the neurotransmitter serotonin, a well-established regulator of egg-laying [13]. Treatment with serotonin causes wildtype worms placed in M9 buffer to spontaneously release their eggs (Fig 2A–2C). *fahd-1(-)* worms on the other hand continued to lay eggs at their “normal” rate upon exposure to 5 or 10 mM exogenous serotonin (Fig 2A and 2B). Interestingly, the egg-laying rate of *fahd-1(-)* worms appeared similar to that observed for wildtype worms treated with 5 mM serotonin. However, addition of 35 mM serotonin, which still stimulated egg-laying in wildtype worms, completely repressed egg-laying in *fahd-1(-)* worms (Fig 2C), suggesting that these animals, at least in certain concentration ranges, can respond to changes in serotonin. For further confirmation of their apparent inability to increase egg-laying upon increased serotonin levels, *fahd-1(-)* worms were exposed to fluoxetine, a serotonin reuptake channel inhibitor. Similar to serotonin itself, this drug effectively stimulated egg-release in wildtype animals, while *fahd-1(-)* worms showed only a mild increase in egg-laying rate (Fig 2D). Together, these observations indicate that *fahd-1* modulates egg-laying by modulating serotonin-signaling.

### Egg-laying behavior in *fahd-1(-)* worms is rescued by exogenous dopamine

**Fig 3:**
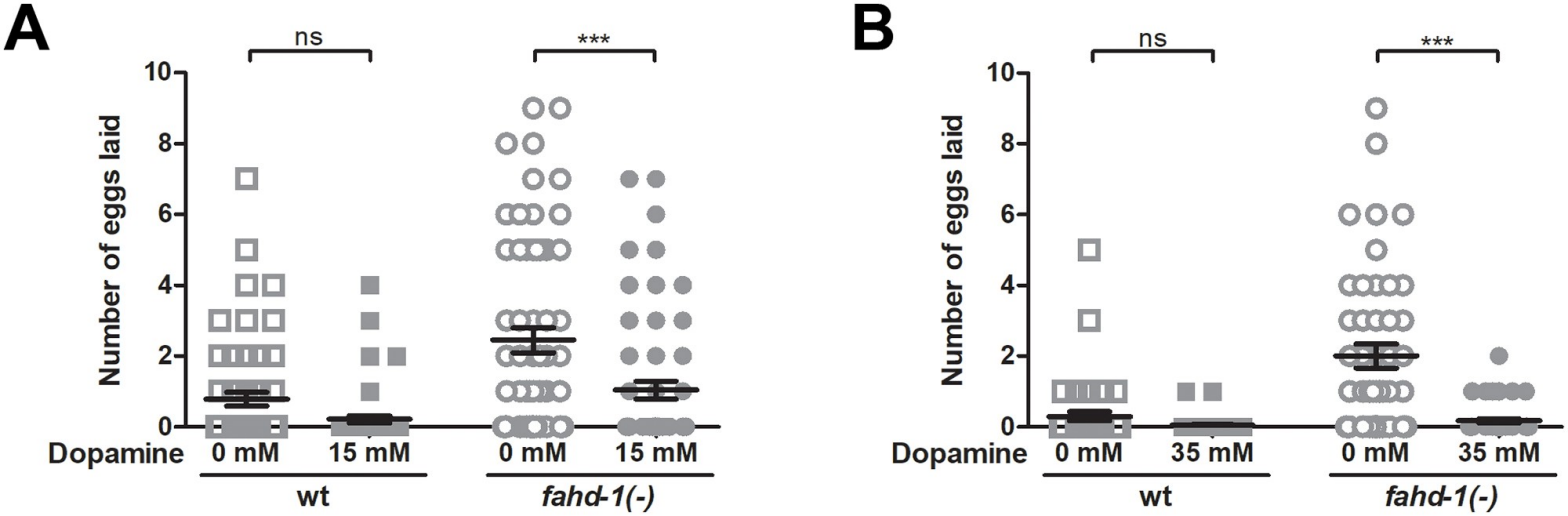
Dopamine effects on egg-laying behavior of *fahd-1*(-) worms. Wild type and *fahd-1(-)* worms were incubated in [A] 15 mM dopamine, or [B] 35 mM dopamine, and the mean number of eggs laid after two hours was determined across at least 45 day one adult worms per strain. Error bars indicate standard errors. Statistical significance was determined by two-way ANOVA with Bonferroni post-tests. *** indicates p<0.001, ns indicates no statistical significance. Data shown is combined from 4–5 independent experiments. See S3 Table for complete statistical analysis.

Contrary to serotonin treatment, dopamine treatment is applied to prevent egg-laying in worms [7]. To investigate the effect of *fahd-1* loss on dopamine signaling, we examined egg-laying of wildtype and *fahd-1*(-) worms in response to 15 and 35 mM dopamine. At 15 μM, dopamine mildly suppressed egg-release in wildtype worms placed in M9 buffer while it reduced the elevated egg-laying rate of *fahd-1*(-) worms to a level similar to that of untreated wildtype animals (Fig 3A). Of note, the dopamine-induced reduction of *fahd-1*(-) egg-laying back to wildtype levels still occurred at the higher dose of 35 mM, which did no longer suppressegg-release in wildtype worms (Fig 3B). In summary, these results suggest that *fahd-1(-)* worms retain sensitivity to dopamine across a wider dose-range than wildtype, and further support the possibility of increased serotonin-levels in these worms.

### Egg-laying in *fahd-1*(-) worms is not sensitive to exogenous levamisole

**Fig 4:**
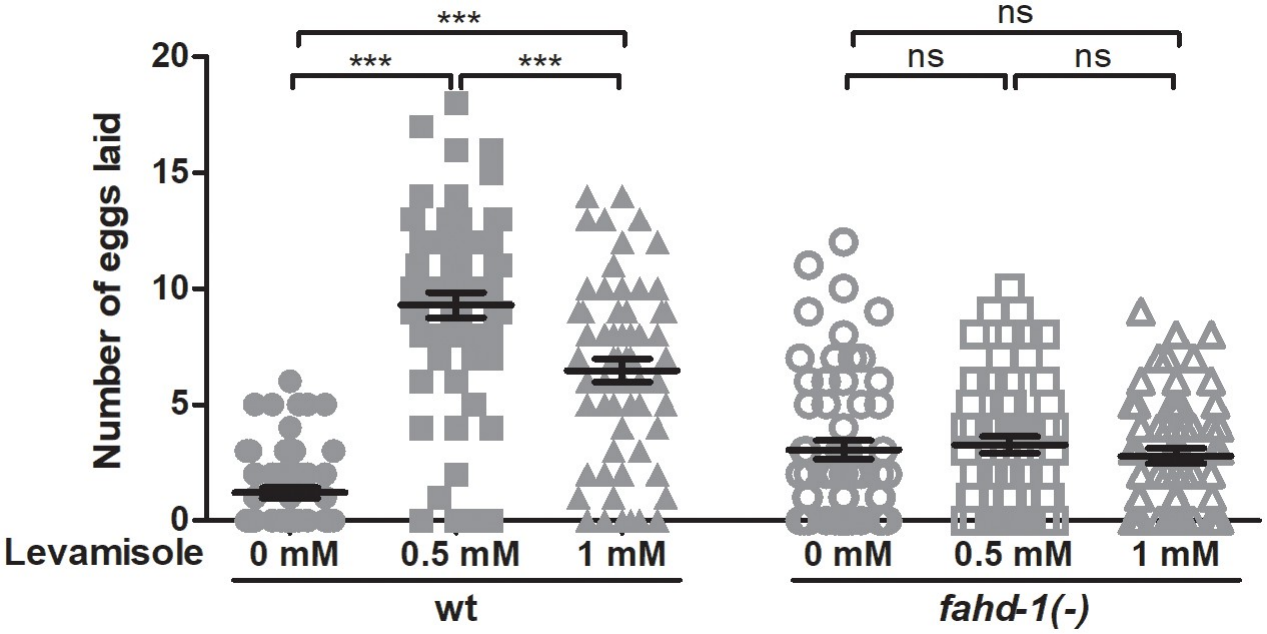
Effects of the acetylcholine agonist levamisole on egg-laying behavior of fahd-1(-) worms. Wild type and *fahd-1(-)* worms were incubated in 0.5 mM and 1 mM levamisole, and the mean number of eggs laid after two hours was determined across at least 55 day one adult worms per strain. Error bars indicate standard errors. Statistical significance was determined by two-way ANOVA with Bonferroni post-tests. *** indicates p<0.001, ns indicates no statistical significance. Data shown is combined from five independent experiments. See S4 Table for complete statistical analysis.

Egg release is also dependent upon regulated release of the neurotransmitter acetylcholine [14]. To ensure that the reduced egg-laying observed in *fahd-1(-)* was due to altered signaling through serotonin, rather than acetylcholine, we treated wildtype and *fahd-1(-)* worms with 0.5 mM and 1 mM of the acetylcholine receptor agonist levamisole (Fig 4). Indeed, while levamisole at both doses increased egg-release in wildtype worms to various extents, it did not alter egg-laying of *fahd-1(-)* worms, indicating that the signaling defect may be located downstream of acetylcholine, consistent with a potential role of serotonin in *fahd-1(-)* egg-laying deficiency.

### Neuronal *fahd-1* modulates egg-laying

**Fig 5:**
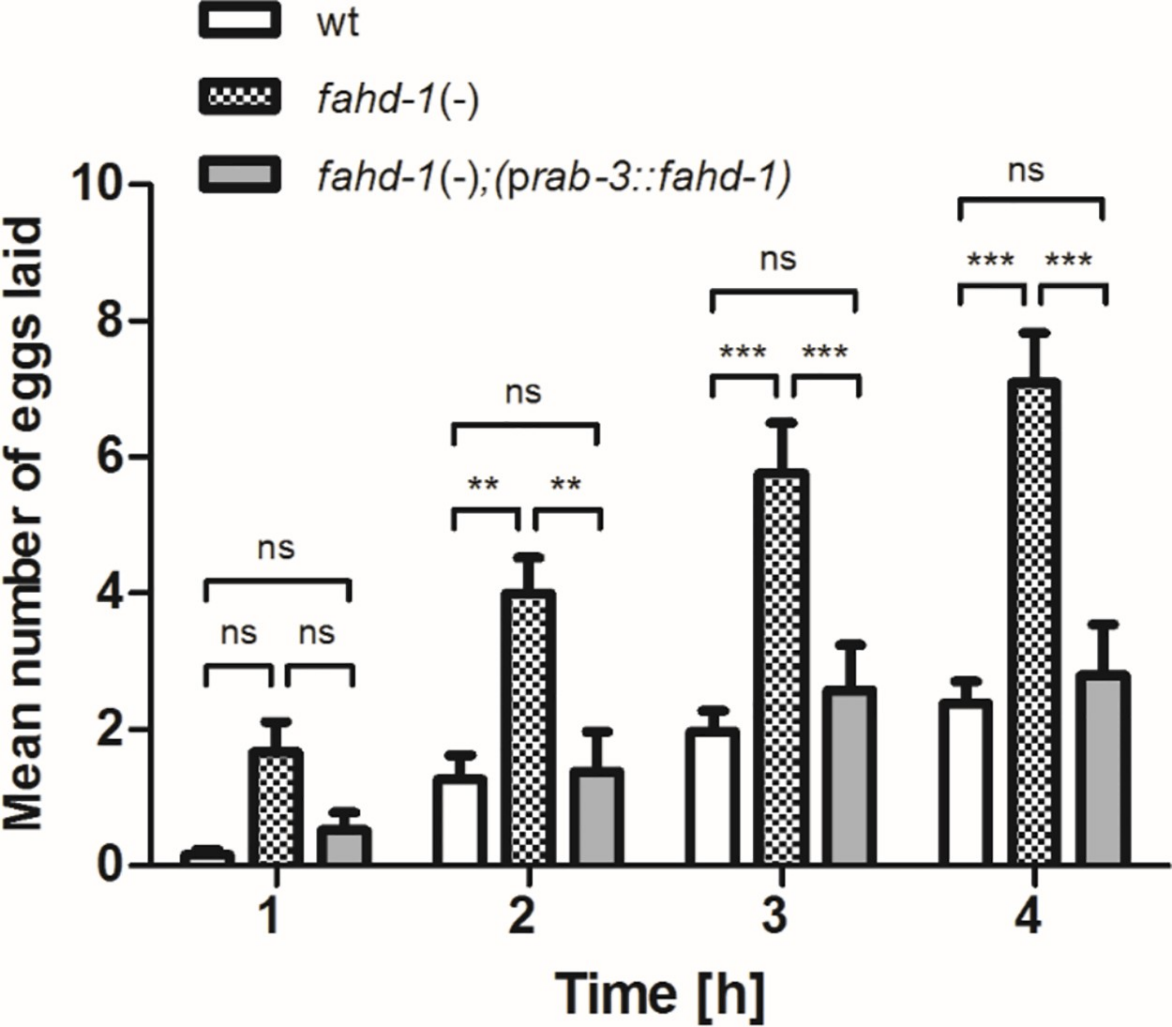
*fahd-1* was re-expressed in *fahd-1*(-) worms under the neuron-specific *rab-3* promoter and the mean number of eggs laid per hour across 60 day one adult worms per strain was determined for four consecutive hours. Error bars indicate standard errors. Statistical significance was determined by two-way ANOVA with Bonferroni post-tests. ** and *** indicate p<0.01 and p<0.001, respectively, ns indicates no statistical significance. Data shown is combined from five independent experiments. See S5 Table for complete statistical analysis.

Having shown that *fahd-1* modulates the worms’s egg-laying response to neurotransmitters, we asked whether normal egg-laying behavior was dependent on *fahd-1’s* function in neurons. Therefore, we compared the egg-laying patterns of neuronally rescued *fahd-1*(-) worms to that of wildtype and *fahd-1*(-) worms after placing them into M9 buffer for 4 h. As observed before [6], under these conditions, wildtype worms retain their eggs while *fahd-1*(-) mutants continue to release them (Fig 5). However, *fahd-1*; [p*rab-3*::*fahd-1*] worms ceased egg-laying under these conditions, just as wildtype. Thus, neuronal *fahd-1* expression is required and sufficient to restore normal egg-laying behavior in *fahd-1(-)* worms.

### *fahd-1* modulates serotonin and dopamine-pathway gene expression

**Fig 6:**
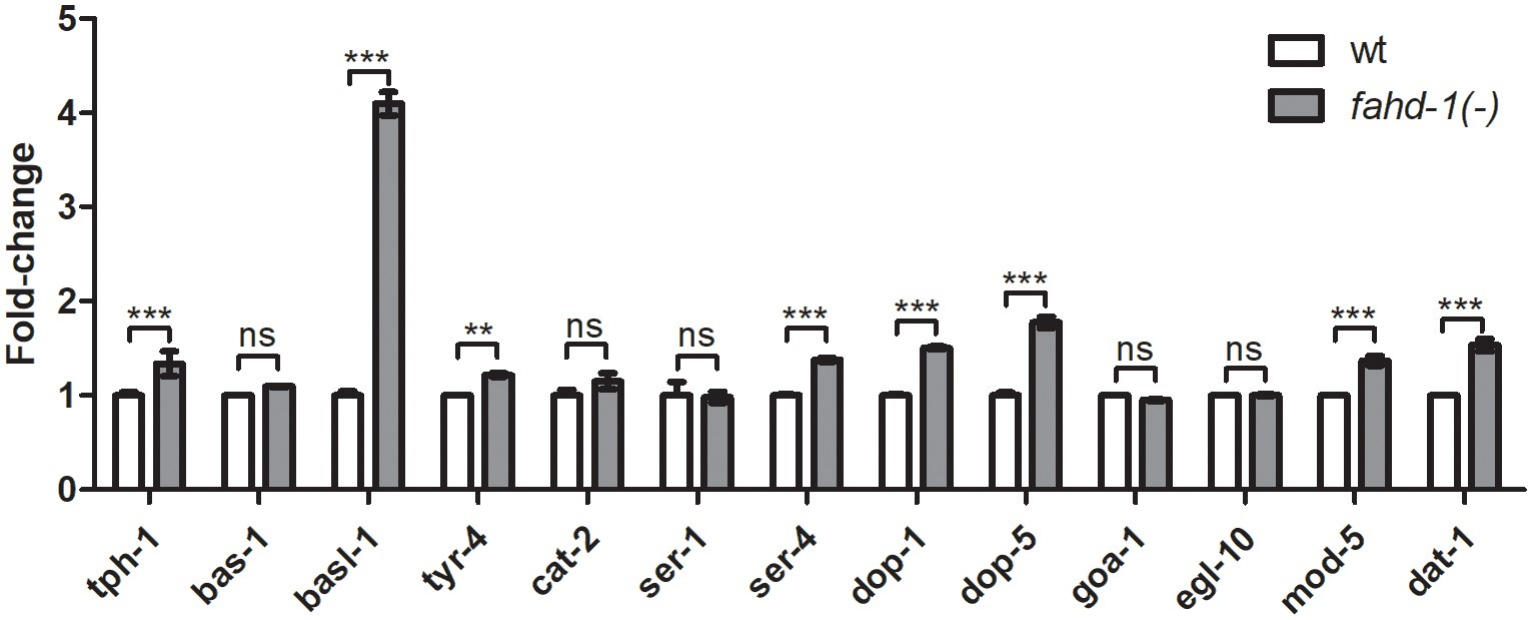
Loss of *fahd-1* increases expression of a subset of serotonin and dopamine pathway genes. [A] Neurotransmitter synthesis in *C*. *elegans*. Adapted from www.wormatlas.org. [B] qPCR analysis to quantify mRNA levels to the serotonin and dopamine genes indicated in wildtype and *fahd-1(-)* worms. Error bars indicate standard deviations of three technical replicates. Statistical significance was determined by two-way ANOVA with Bonferroni post-tests. ** and *** indicate p<0.01 and p<0.001, respectively, ns indicates no statistical significance. Data shown is representative for three independent experiments. See S6 Table for complete statistical analysis.

Given the apparent function of *fahd-1* in serotonin/dopmain regulated egg-laying, we analyzed the effect of *fahd-1* loss on the expression of selected serotonin and dopamine pathway genes by qPCR. As our results provide evidence for elevated serotonin levels in *fahd-1(-)* worms, we specifically focused on genes involved in serotonin and dopamine biosynthesis (Fig 6). All of the dopamine synthesis and -signaling genes (*cat-2*, a tyrosine hydroxylase; *egl-10*, a regulator of G-signaling protein, *tyr-4*, a tyrosinase, dat-1, a sodium-dependent dopamine transporter) and most of the serotonin synthesis genes (*tph-1*, a tryptophan hydroxylase; *bas-1*, an aromatic amino acid decarboxylase; *basl-1*, an aromatic amino acid decarboxylase like protein) displayed constitutive expression in wildtype worms. In *fahd-1(-)* worms, a relatively small but statistically significant increase in expression of *tph-1*, *dat-1*, and *tyr-4* was observed. Moreover, *fahd-1* deficiency dramatically increased mRNA-levels of the *bas*-like gene *basl-1*. One serotonin and both dopamine receptors (*ser-4*, *dop-1*, and *dop-5*, respectively), as well as the serotonin-reuptake channel (*mod-5*) were also moderately upregulated at the mRNA-level upon *fahd-1* loss. Together, these gene expression data are consistent with the model that serotonin biosynthesis and sensitivity are increased in *fahd-1*(-) worms, and this elevation translates into increased activity of serotonin-induced processes, as it cannot be compensated for by a concomitant increase in serotonin re-uptake and dopamine sensitivity.

## Discussion

In the present work, we address potential mechanisms underlying the impaired egg-laying pattern in *fahd-1(-)* worms. Our findings indicate that *fahd-1* modulates these behaviors at least in part through its action in neurons. Re-introducing *fahd-1* in neurons partially rescued the locomotion deficit and fully restored a wildtype-like egg-laying pattern of *fahd-1(-)* worms. On the other hand, *fahd-1* overexpression under its native promoter in *fahd-1*(+) animals impaired locomotion to a similar extent than *fahd-1* loss. Importantly, *fahd-1* physiologically is not only expressed in neurons but also in other large tissues such as muscle and intestine, as well as in pharynx, vulva and canal cell [6]. Therefore, expression of *fahd-1* in various tissues, trough cell-autonomous effects and/or through cell non-autonomous effects of *fahd-1* in neurons, as well as an appropriate expression level, appears to be required for a fully wildtype phenotype.

While neuronal expression of *fahd-1* is sufficient to restore normal egg-laying behavior to *fahd-1*(-) worms, it does not rule out the possibility that *fahd-1* in other tissues also plays a role. Egg release in nematodes is regulated by the neurotransmitters serotonin and dopamine [8]. We observed that treatment with serotonin failed to stimulate egg-laying in *fahd-1*(-) worms, while exogenous dopamine suppressed the excessive egg release of *fahd-1(-)* worms back to wild type levels, indicating that *fahd-1* modulates signaling through these neurotransmitters.

More specifically, our results raise the possibility that *fahd-1* deficiency causes an imbalance between serotonin and dopamine synthesis. Importantly, exposure to high concentrations of both neurotransmitters completely inhibited egg-laying indicating that *fahd-1(-)* worms are in principle able to respond to them (S1 Fig). Consistent with this model, qPCR analysis of *tph-1*, *bas-1* and *cat-2*, provides evidence for the biosynthesis of serotonin being favored over that of dopamine since the rate-limiting serotonin-biosynthetic enzyme *tph-1* is overexpressed, while expression levels of the rate-limiting dopamine-biosynthetic enzyme *cat-2* remain unchanged upon *fahd-1* loss. Of note, we observed a strong induction of *basl-1*, a currently uncharacterized gene/protein with extensive sequence similarity to *bas-1*, which, however, lacks some amino acids that are predicted to be important for *bas-1* catalytic activity (wormbase.org). Thus, it is currently unclear if and eventually how, *basl-1* may modulate serotonin biosynthesis.

Finally, we observed upregulation of dopamine receptors and reuptake channels (*dop-1*, *dop-5*, *dat-1*) and serotonin reuptake channel (*mod-5*) on the mRNA level in *fahd-1(-)* worms. In light of the apparently excess synthesis of serotonin, this induction may reflect a compensatory response with the goal of partially restoring the balance between serotonin and dopamine signaling.

In mammals, the oxaloacetate decarboxylase FAHD1 plays a critical role in fine-tuning the concentration of TCA cycle-related metabolites, such as oxaloacetate (OAA) and pyruvate [3, 5]. Its homology to human FAHD1 suggests that the nematode FAHD-1 protein may function as an oxaloacetate decarboxylase as well. How this putative enzymatic activity relates to *fahd-1*’s effects on transcriptional regulation of serotonin signaling observed here remains elusive. Recent studies have proposed a cross-talk between metabolic pathways in the mitochondria and epigenetic mechanisms in the nucleus, such as posttranslational modifications of histones [15]. It is conceivable that perturbations of the TCA cycle flux, resulting from *fahd-1* deletion in nematodes, may lead to changes in the size of the nucleo-cytosolic pool of acetyl-CoA [16], which would have a direct impact on histone acetylation [17]. One may speculate that depletion of *fahd-1* in *C*.*elegans* leads to changes in the acetylation state of histones involved in the regulation of specific genes, including several genes encoding serotonin synthesis and dopamine receptor proteins, as described in this communication. More work will be required to investigate in detail the mechanism by which deletion of the *fahd-1* gene affects transcriptional regulation of the serotonin signaling pathway in nematodes.

## Supporting information

S1 Fig (DOCX)

S2 Fig (DOCX)

S1 Table (DOCX)

S2 Table (DOCX)

S3 Table (DOCX)

S4 Table (DOCX)

S5 Table (DOCX)

S6 Table (DOCX)

S7 Table (DOCX)

S8 Table (DOCX)

S9 Table (DOCX)
