# Supplementary material for: Modulation of serotonin signaling by the putative oxaloacetate decarboxylase FAHD-1 in Caenorhabditis elegans
Source: PLoS One. 2019 Aug 14;14(8):e0220434. doi: 10.1371/journal.pone.0220434 (PMC6693844; doi:10.1371/journal.pone.0220434)
Supplement: S1 Fig — (DOCX) [file pone.0220434.s001.docx]

# Supporting Information


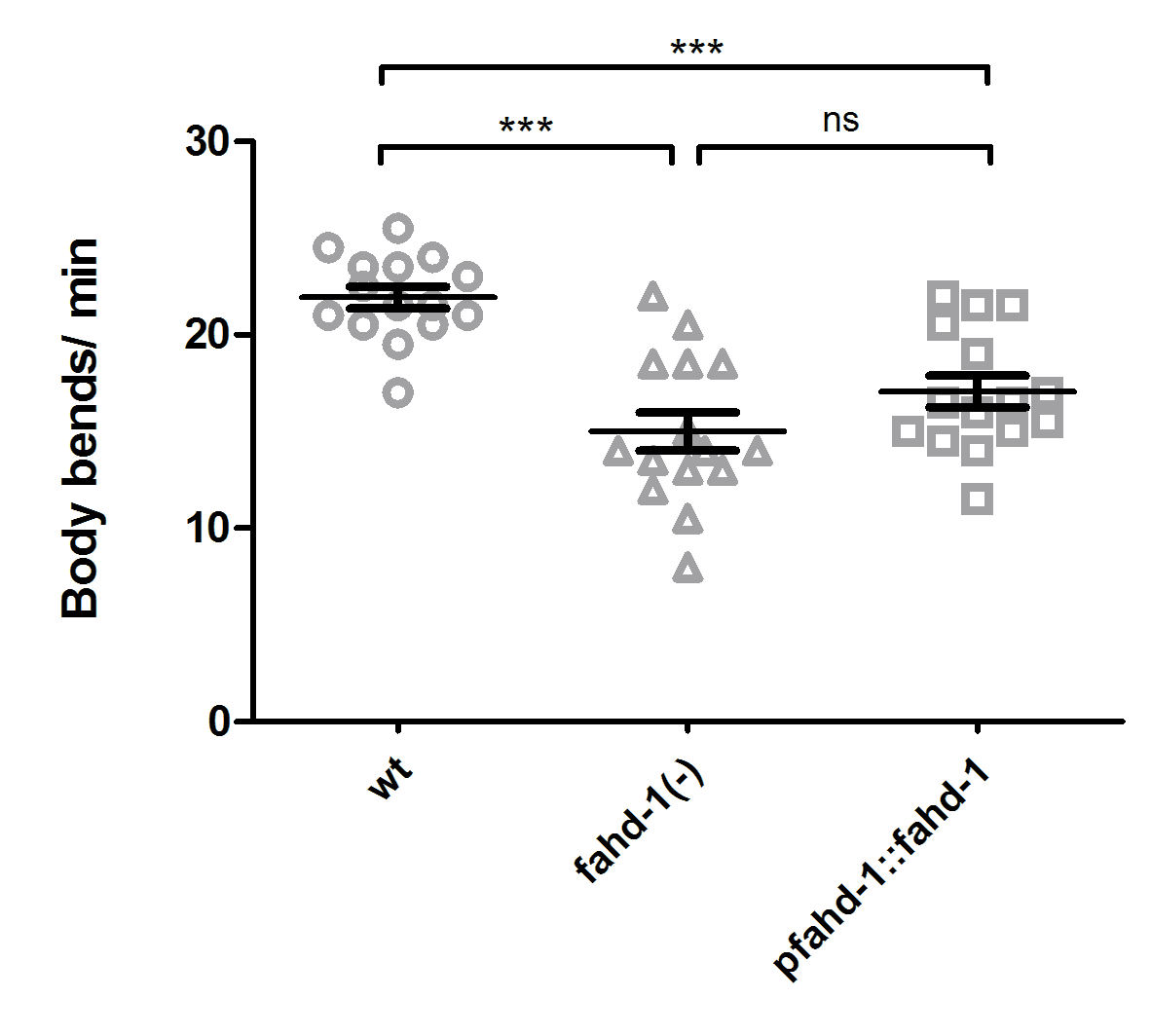


**S1 Fig. *fahd-1* overexpression in *fahd-1(+)* background suppresses body-bending**

*fahd-1* was re-expressed under the control of its native promoter in *fahd-1(+)* worms, and the mean number of body bends per minute across at least 45 day one adult worms per strain was determined. Error bars indicate standard errors. Statistical significance was determined by one-way ANOVA with Bonferroni post-tests. *** indicates p<0.001, ns indicates no statistical significance. Data shown is representative for two independent experiments. See S8 Table for complete statistical analysis.
