# Supplementary material for: Modulation of serotonin signaling by the putative oxaloacetate decarboxylase FAHD-1 in Caenorhabditis elegans
Source: PLoS One. 2019 Aug 14;14(8):e0220434. doi: 10.1371/journal.pone.0220434 (PMC6693844; doi:10.1371/journal.pone.0220434)
Supplement: S2 Fig — (DOCX) [file pone.0220434.s002.docx]

# Supporting Information

**
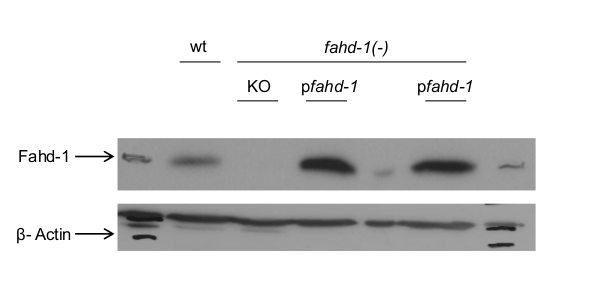
**

**S2 Fig. Western Blot analysis of FAHD-1 protein expression**

FAHD-1 expression was analyzed by Western Blot in 30 µg total protein lysate from the strains indicated. The *fahd-1(-);* *[pfahd-1::fahd-1]* strain was analyzed in biological duplicates. The housekeeping gene β-actin served as a loading control.
