# Supplementary material for: Modulation of serotonin signaling by the putative oxaloacetate decarboxylase FAHD-1 in Caenorhabditis elegans
Source: PLoS One. 2019 Aug 14;14(8):e0220434. doi: 10.1371/journal.pone.0220434 (PMC6693844; doi:10.1371/journal.pone.0220434)
Supplement: S1 Table — (DOCX) [file pone.0220434.s003.docx]

**S1 Table: Body bend assay statistics.**

Accompanies Fig. 1. p-values are from one-way ANOVA with Bonferroni post-tests.

*Panel A*

|  | **wt** | ***fahd-1*(-)** | ***fahd-1*(-)*;*[p*fahd-1::fahd-1*]** |
| --- | --- | --- | --- |
| **Mean** | 26.92 | 18.10 | 20.80 |
| **SEM** | 0.44 | 0.58 | 0.84 |
| **N** | 50 | 50 | 50 |
| **p-value vs. wt** |  | <0.001 | <0.001 |
| **p-value vs. *fahd-1(-)*** | <0.001 |  | <0.05 |

*Panel B*

|  | **wt** | ***fahd-1*(-)** | ***fahd-1*(-)*;*[p*fahd-1::fahd-1*]** |
| --- | --- | --- | --- |
| **Mean** | 27.31 | 17.94 | 20.29 |
| **SEM** | 0.58 | 0.73 | 0.71 |
| **N** | 35 | 35 | 35 |
| **p-value vs. wt** |  | <0.001 | <0.001 |
| **p-value vs. *fahd-1(-)*** | <0.001 |  | <0.05 |
