# Supplementary material for: Modulation of serotonin signaling by the putative oxaloacetate decarboxylase FAHD-1 in Caenorhabditis elegans
Source: PLoS One. 2019 Aug 14;14(8):e0220434. doi: 10.1371/journal.pone.0220434 (PMC6693844; doi:10.1371/journal.pone.0220434)
Supplement: S2 Table — (DOCX) [file pone.0220434.s004.docx]

**S2 Table: Exposure to serotonin assay statistics.**

Accompanies Fig. 2. p-values are from two-way ANOVA with Bonferroni post-tests.

*Panel A*

*Combined data from 5 independent experiments, each comprising 10-12 worms per strain.*

| **Genetic background** | **Serotonin [mM]** | **Mean** | **SEM** | **N** | **p-Value**  **0 vs. 5 mM** |
| --- | --- | --- | --- | --- | --- |
| **wt** | 0 | 0.64 | 0.16 | 58 |  |
|  | 5 | 3.02 | 0.48 | 56 | *** |
| ***fahd-1(-)*** | 0 | 2.45 | 0.37 | 58 |  |
|  | 5 | 2.45 | 0.38 | 58 | ns |

*Panel B*

*Combined data from 5 independent experiments, each comprising 12 worms per strain.*

| **Genetic background** | **Serotonin [mM]** | **Mean** | **SEM** | **N** | **p-Value**  **0 vs. 10 mM** |
| --- | --- | --- | --- | --- | --- |
| **wt** | 0 | 0.30 | 0.13 | 60 |  |
|  | 10 | 7.80 | 0.76 | 60 | *** |
| ***fahd-1(-)*** | 0 | 2.12 | 0.36 | 60 |  |
|  | 10 | 2.00 | 0.31 | 60 | ns |

*Panel C*

*Combined data from 5 independent experiments, each comprising 11-12 worms per strain.*

| **Genetic background** | **Serotonin [mM]** | **Mean** | **SEM** | **N** | **p-Value**  **0 vs. 35 mM** |
| --- | --- | --- | --- | --- | --- |
| **wt** | 0 | 0.42 | 0.15 | 60 |  |
|  | 35 | 4.97 | 0.63 | 60 | *** |
| ***fahd-1(-)*** | 0 | 1.54 | 0.33 | 59 |  |
|  | 35 | 0.28 | 0.10 | 60 | * |

*Panel D*

*Combined data from 3 independent experiments, with #1 and #2 comprising 21-24 and #3 comprising 13 worms per strain.*

| **Genetic background** | **Fluoxetine [mg/ml]** | **Mean** | **SEM** | **N** | **p-Value**  **0 vs. 0.5 mg/ml** |
| --- | --- | --- | --- | --- | --- |
| **wt** | 0 | 0.24 | 0.08 | 59 |  |
|  | 0.5 | 9.33 | 0.85 | 58 | *** |
| ***fahd-1(-)*** | 0 | 2.23 | 0.35 | 60 |  |
|  | 0.5 | 3.35 | 0.49 | 57 | ns |
