# Supplementary material for: Modulation of serotonin signaling by the putative oxaloacetate decarboxylase FAHD-1 in Caenorhabditis elegans
Source: PLoS One. 2019 Aug 14;14(8):e0220434. doi: 10.1371/journal.pone.0220434 (PMC6693844; doi:10.1371/journal.pone.0220434)
Supplement: S3 Table — (DOCX) [file pone.0220434.s005.docx]

**S3 Table: Exposure to dopamine assay statistics.**

Accompanies Fig. 3. p-values are from two-way ANOVA with Bonferroni post-tests.

*Panel A*

*Combined data from 5 independent experiments, each comprising 11-12 worms per strain.*

| **Genetic background** | **Dopamine [mM]** | **Mean** | **SEM** | **N** | **p-Value**  **0 vs. 15 mM** |
| --- | --- | --- | --- | --- | --- |
| **wt** | 0 | 0.78 | 0.19 | 59 |  |
|  | 15 | 0.21 | 0.10 | 57 | ns |
| ***fahd-1(-)*** | 0 | 2.45 | 0.36 | 60 |  |
|  | 15 | 1.03 | 0.25 | 58 | *** |

*Panel B*

*Combined data from 4 independent experiments, each comprising 11-12 worms per strain.*

| **Genetic background** | **Dopamine [mM]** | **Mean** | **SEM** | **N** | **p-Value**  **0 vs. 35 mM** |
| --- | --- | --- | --- | --- | --- |
| **wt** | 0 | 0.30 | 0.13 | 47 |  |
|  | 35 | 0.04 | 0.03 | 47 | ns |
| ***fahd-1(-)*** | 0 | 2.00 | 0.34 | 47 |  |
|  | 35 | 0.17 | 0.06 | 48 | *** |
