# Supplementary material for: Modulation of serotonin signaling by the putative oxaloacetate decarboxylase FAHD-1 in Caenorhabditis elegans
Source: PLoS One. 2019 Aug 14;14(8):e0220434. doi: 10.1371/journal.pone.0220434 (PMC6693844; doi:10.1371/journal.pone.0220434)
Supplement: S4 Table — (DOCX) [file pone.0220434.s006.docx]

**S4 Table:** **Exposure to levamisole assay statistics.**

Accompanies Fig. 4. p-values are from two-way ANOVA with Bonferroni post-test. Data shown is combined from 5 independent experiments, each comprising 11-12 worms per strain.

| **Genetic background** | **Levamisole [mM]** | **Mean** | **SEM** | **N** | **p-Value**  **vs. 0 mM** | **p-Value**  **vs. 0.5 mM** |
| --- | --- | --- | --- | --- | --- | --- |
| **wt** | 0 | 1.20 | 0.22 | 59 |  |  |
|  | 0.5 | 9.30 | 0.55 | 60 | *** |  |
|  | 1 | 6.48 | 0.51 | 60 | *** | *** |
| ***fahd-1(-)*** | 0 | 3.07 | 0.41 | 60 |  |  |
|  | 0.5 | 3.27 | 0.37 | 59 | ns |  |
|  | 1 | 2.80 | 0.32 | 60 | ns | ns |
