# Supplementary material for: Modulation of serotonin signaling by the putative oxaloacetate decarboxylase FAHD-1 in Caenorhabditis elegans
Source: PLoS One. 2019 Aug 14;14(8):e0220434. doi: 10.1371/journal.pone.0220434 (PMC6693844; doi:10.1371/journal.pone.0220434)
Supplement: S5 Table — (DOCX) [file pone.0220434.s007.docx]

**S5 Table: Egglaying assay**

Accompanies Fig. 5. p-values are from two-way ANOVA withBonferroni post-tests. Data shown is combined from 5 independent experiments, each comprising

|  | **wt** | | | ***fahd-1(-)*** | | | **fahd-1(-);[p_rab-3::fahd-1]** | | | **p-Values** | | |
| --- | --- | --- | --- | --- | --- | --- | --- | --- | --- | --- | --- | --- |
| **[h]** | **Mean** | **SEM** | **N** | **Mean** | **SEM** | **N** | **Mean** | **SEM** | **N** | **wt/**  **fahd-1(-)** | **wt/**  **fahd-1(-);[prab-3::fahd-1]** | **fahd-1(-)/**  **fahd-1(-);[prab-3::fahd-1]** |
| 1 | 0.16 | 0.07 | 60 | 1.67 | 0.44 | 60 | 0.52 | 0.26 | 60 | > 0.05 | > 0.05 | > 0.05 |
| 2 | 1.27 | 0.35 | 60 | 4.00 | 0.53 | 60 | 1.38 | 0.59 | 60 | <0.01 | > 0.05 | <0.01 |
| 3 | 1.97 | 0.31 | 60 | 5.76 | 0.74 | 60 | 2.58 | 0.65 | 60 | <0.001 | > 0.05 | <0.001 |
| 4 | 2.38 | 0.32 | 60 | 7.08 | 0.73 | 60 | 2.79 | 0.75 | 60 | <0.001 | > 0.05 | <0.001 |
