# Supplementary material for: Modulation of serotonin signaling by the putative oxaloacetate decarboxylase FAHD-1 in Caenorhabditis elegans
Source: PLoS One. 2019 Aug 14;14(8):e0220434. doi: 10.1371/journal.pone.0220434 (PMC6693844; doi:10.1371/journal.pone.0220434)
Supplement: S6 Table — (DOCX) [file pone.0220434.s008.docx]

# Supporting Information

**S6 Table: qPCR statistics.**

Accompanies Fig. 6. p-values are from two-way ANOVA with Bonferroni post-tests.

|  | ***fahd-1(-)* vs. wt** | | |
| --- | --- | --- | --- |
|  | **Fold-change** | | **p-value** |
| **Target gene** | **Mean** | **SD** |  |
| ***tph-1*** | 1.34 | 0.13 | <0.001 |
| ***bas-1*** | 1.09 | 0.01 | > 0.05 |
| ***basl-1*** | 4.10 | 0.12 | <0.001 |
| ***tyr-4*** | 1.22 | 0.02 | <0.01 |
| ***cat-2*** | 1.15 | 0.08 | > 0.05 |
| ***ser-1*** | 0.98 | 0.06 | > 0.05 |
| ***ser-4*** | 1.38 | 0.02 | <0.001 |
| ***dop-1*** | 1.51 | 0.02 | <0.001 |
| ***dop-5*** | 1.78 | 0.06 | <0.001 |
| ***goa-1*** | 0.95 | 0.01 | > 0.05 |
| ***egl-10*** | 1.00 | 0.01 | > 0.05 |
| ***mod-5*** | 1.37 | 0.05 | <0.001 |
| ***dat-1*** | 1.53 | 0.07 | <0.001 |
