# Supplementary material for: Modulation of serotonin signaling by the putative oxaloacetate decarboxylase FAHD-1 in Caenorhabditis elegans
Source: PLoS One. 2019 Aug 14;14(8):e0220434. doi: 10.1371/journal.pone.0220434 (PMC6693844; doi:10.1371/journal.pone.0220434)
Supplement: S7 Table — (DOCX) [file pone.0220434.s009.docx]

Supporting Information

**S7 Table: List of primers used in this study for qPCR or genotyping**

***egl-10*** fw: TGAAGCAGCGGATCATATCTAC; rev: GCGATGCTTTACGAGCAATCA;

***goa-1*** fw: TCGATGTGGGAGGTCAAAGA ; rev: CGTGCATTCGGTTTGTTGTCT;

***basl-1*** fw: AAAGAAAGACCGTGTCCCTCG; rev: GAGACGCTCGTTGTCAGAGT;

***cat-2*** fw: GGCGTTAGAGTTCAAGTTTGGT rev: CCGCTGTCAAAACCTTCTCC;

***tph-1*** fw: AGTGACAATCGCATGGAGGA; rev: TACGAGTTGGTGTGAAGAGTTGT;

***cdc-42*** fw: CGCCGTCACAGTAATGATCG; rev: CTCCTGTTGTGGTGGGTCG;

***tyr-4*** fw: AGCATCTGGGGAATGTTCGAG; rev: AGTGCAATCATCAGCCAGTCG;

***bas-1*** fw: GAAAAATAGCACCAACGCGGA; rev: CGGCGAGTTCAAAGATGACG;

***dat-1*** fw: GACATTGCTCTTTCCCTCTTCG; rev: TTTTGCCATCCGGGTAGAGTC;

***mod-5*** fw: CCCGTTGTTTCGAGGAATCG; rev: AATAGACGGCTTGAGCGATGA;

***dop-1*** fw : GTCCCCACAAGATCCTCACA; rev: AATAACACCCAATGTAAGACGTGC;

***dop-3*** fw: TGGAAGAGAACGATGAATGCG; rev : CTTCTTGCTCGCTCTCGTTG;

***ser-1*** fw GAAGAAGATCTCCCTCCCATCAT; rev: GTGGTTGATGCCTCTGTCGT;

***ser-4*** fw: CAGAATAGGACCCCGAAGCG; rev: CAGGACTCTGTCACTAAACCCC;

***fahd-1*** F1 fw: CATGTCTTCACTCGCTGGAT; R1 rev: GATCATCACATCGGTACGAC;

F2: fw: CATTGGAGGTTACACTGTCG
