# Supplementary material for: Modulation of serotonin signaling by the putative oxaloacetate decarboxylase FAHD-1 in Caenorhabditis elegans
Source: PLoS One. 2019 Aug 14;14(8):e0220434. doi: 10.1371/journal.pone.0220434 (PMC6693844; doi:10.1371/journal.pone.0220434)
Supplement: S8 Table — (DOCX) [file pone.0220434.s010.docx]

Supporting Information

**S8 Table*:* Statistics of body bend assay with *fahd-1* full body overexpression strain *pfahd-1::fahd-1***

Accompanies S2 Fig. P-values are from one-way ANOVA.

|  | **wt** | ***fahd-1*(-)** | ***pfahd-1::fahd-1*** |
| --- | --- | --- | --- |
| **mean** | 21.93 | 15.00 | 17.07 |
| **SD** | 2.18 | 3.85 | 3.15 |
| **n** | ≥ 40 | ≥ 40 | ≥ 40 |
| **p-value vs. wt** |  | <0.001 | <0.001 |
| **p-value vs. fahd-1(-)** | <0.001 |  | >0.05 |

# Supporting Information

**S9 Table: *C. elegans* strains used in this study**

- N2 anchestral wild type
- HMT059: *fahd-1(tm5005*) *III*
- HMT060: *fahd-1(tm5005*) *III*; *thmEx001 [*p*fahd-1::fahd-1 + pL4040 (*p*myo-2::gfp)]*
- HMT061: *fahd-1(tm5005*) *III*; *thmEx002 [*p*rab-3::fahd-1 + pL4040 (*p*myo-2::gfp)]*
- HMT062: *fahd-1(tm5005*) *III*; *thmEx003 [*p*myo-3::fahd-1 + pL4040 (*p*myo-2::gfp)]*
- HMT067: *thmEx001 [**pfahd-1::fahd-1 +* *pL4040 (pmyo-2::**gfp)]*
